# Fourmidable: a database for ant genomics

**DOI:** 10.1186/1471-2164-10-5

**Published:** 2009-01-06

**Authors:** Yannick Wurm, Paolo Uva, Frédéric Ricci, John Wang, Stephanie Jemielity, Christian Iseli, Laurent Falquet, Laurent Keller

**Affiliations:** 1 Department of Ecology and Evolution, Biophore, University of Lausanne, CH-1015 Lausanne, Switzerland; 2 Istituto di Ricerche di Biologia Molecolare, Merck Research Laboratories, 00040 Pomezia, Rome, Italy; 3 Institut for Infectious Diseases, University of Bern, CH-3010 Bern, Switzerland; 4 Ludwig Institute for Cancer Research, CH-1015 Lausanne, Switzerland; 5 Swiss Institute of Bioinformatics, CH-1015 Lausanne, Switzerland

## Abstract

**Background:**

Fourmidable is an infrastructure to curate and share the emerging genetic, molecular, and functional genomic data and protocols for ants.

**Description:**

The Fourmidable assembly pipeline groups nucleotide sequences into clusters before independently assembling each cluster. Subsequently, assembled sequences are annotated via Interproscan and BLAST against general and insect-specific databases. Gene-specific information can be retrieved using gene identifiers, searching for similar sequences or browsing through inferred Gene Ontology annotations. The database will readily scale as ultra-high throughput sequence data and sequences from additional species become available.

**Conclusion:**

Fourmidable currently houses EST data from two ant species and microarray gene expression data for one of these. Fourmidable is publicly available at

## Background

Ants are important model species for sociobiology and behavioral ecology [1]. Life in an ant colony is marked by cooperation, but it also entails conflicts. Both aspects have been studied extensively to understand the prerequisites for social behavior and to test the kin selection theory (e.g. [2,3]). New molecular and genomic techniques are making it possible to identify the genes underlying social behavior in ants and other social insects [4-11] as well as other fascinating aspects of social life including self-organization, life-history evolution, division of labor, and developmental plasticity [12-15]. The extraordinary complexity and vast information content generated by modern genomic techniques can be overwhelming. To take full advantage of such techniques requires appropriate bioinformatics tools.

To provide a central repository for the emerging ant genomic data, we developed Fourmidable, a web-accessible, user-friendly tool. Fourmidable currently provides detailed assembly and annotation of nucleic acid sequences from ants, a repository for ant microarray experiments and a platform to share ant-specific molecular biology protocols.

## Construction and content

Formidable contains publicly available sequence and gene expression data for ants and analyses of these data (summarized in Table 1).

**Table 1:** Data Content in Fourmidable (October 2008)

***Solenopsis invicta:***
28,006 input sequences including:
• Tracefiles from 21,715 ESTs (some were multiply sequenced)
• 1,496 additional ESTs and mRNA sequences from GenBank
12,859 putative transcripts:
• 4,958 contigs
• 7,263 singlets
Sequence annotation:
• 14,222 annotating GO terms on 2,818 putative transcripts
• 599 Interproscan annotations
• Blast comparisons against the non-redundant protein database, as well as proteomes and genomes of *Apis mellifera*, *Anopheles gambiae *and *Drosophila melanogaster*.
Microarray data:
• Two public experiments
• 66 hybridizations
***Lasius niger:***
709 input sequences which are:
• Tracefiles from 615 EST clones
403 putative transcripts:
• 147 contigs
• 256 singlets
21 Interproscan annotations
***General:***
8 molecular biology protocols

### Computation and Database Design

Fourmidable analyses are carried out via custom Perl scripts and publicly available software. Annotation and assembly information is stored in a MySQL database while sequences and BLAST [16] results are kept in indexed text files for rapid retrieval while limiting database size. Data are stored separately for different species [see Additional file 1]. Computationally intensive tasks are parallelized on the Vital-IT high-performance computing cluster [[17], Additional file 2]. Fourmidable should thus readily handle large amounts of additional data. Data is accessible to web users via a PHP/Apache-based interface hosted by the Swiss Institute of Bioinformatics.

### Nucleotide Sequence Data Preparation, Assembly, and Annotation

Fourmidable currently processes nucleotide sequences for the red fire ant *Solenopsis invicta *and the black garden ant *Lasius niger*. When available, raw .ab1 or .scf trace files are converted via Phred [18] to FASTA nucleotide and quality score files. Additional sequences for which trace files cannot be obtained are downloaded from Genbank; quality score files are generated for these sequences with an arbitrary Phred quality score of 25. All input sequences are cleaned using Lucy [19], DUST [Tatusov and Lipman, unpublished], RepeatMasker [20] and CrossMatch [21] to respectively remove low-quality regions and sequences, low-complexity regions, interspersed repeats, and sequences from bacteria, organelles or cloning vectors. Cleaned sequences are then compared via reciprocal BLAST [16], and subsequently similar sequences are grouped into clusters. Within each cluster, sequences are independently assembled via CAP3 [22]. This circumvents memory constraints that CAP3 would face if attempting a global assembly with large numbers of sequences. The output from clustering and CAP3 assemblies are contigs (each is the consensus of several assembled sequences) and singlets (sequences that did not assemble with others). All sequences are subsequently annotated as follows.

All sequences are compared to the non-redundant protein database [23] via BLASTX as well as to several insect-specific databases via TBLASTX, BLASTX and BLASTN. Gene Ontology (GO) annotation of the strongest of the top five BLASTX hits to the non-redundant protein database is carried over to ant sequences.

To determine possible peptide sequences, we compute all six possible translations of transcriptome sequences with the potential to encode sequences longer than 30 amino acids. We do not use an *ab initio *gene prediction program such as ESTScan [24] because the sensitivity of such programs is limited by the absence of solid training data for ants. Instead, all potential open reading frames are annotated via Interproscan [25]. Some Interproscan hits directly provide GO annotation of ant sequences, complementing the BLASTX-inferred GO annotation mentioned above.

The BLAST, Interproscan and GO annotations are updated every two months or when new sequence data is added to the assembly pipeline.

At several steps during this assembly and annotation pipeline, bioinformatics software was run with parameters that differed from default parameters [see Additional file 3], as determined by using reduced test datasets.

### Gene Expression

Fourmidable is linked to the GEDAI gene expression database [Robin Liechti, unpublished]. Storage and simple analysis using Bioconductor packages [26] is possible for data from single-color and two-color spotted microarrays, as well as for Affymetrix and Illumina microarrays.

## Utility and discussion

As of October 2008, Fourmidable contains nucleotide sequence data for the fire ant *S. invicta *and the black garden ant *L. niger *as well as gene expression data for *S. invicta*. Currently accessible data are summarized in Table 1. Sequencing, gene expression profiling, and genotyping data are rapidly expanding and will be added as they become publicly available. Fourmidable's home page [27] centralizes links and search facilities to access Fourmidable's data and tools.

### Sequence Search

There are several manners of accessing sequence information in Fourmidable. First, single sequences can be searched by species as well as by partial identifiers for input sequences or assembled contigs. Second, lists of identifiers can be used for searching. Third, user-supplied sequence data can be used for BLAST similarity searches against sequences in Fourmidable. Finally, users can navigate inferred Gene Ontology annotations for biological processes, cellular components, and molecular functions using the AmiGO browser [28]. The first two search manners result in tables as described below. BLAST searching and GO browsing produce lists of sequence identifiers that can be used as inputs for the first two search manners.

### Sequence Information

**Figure 1:**
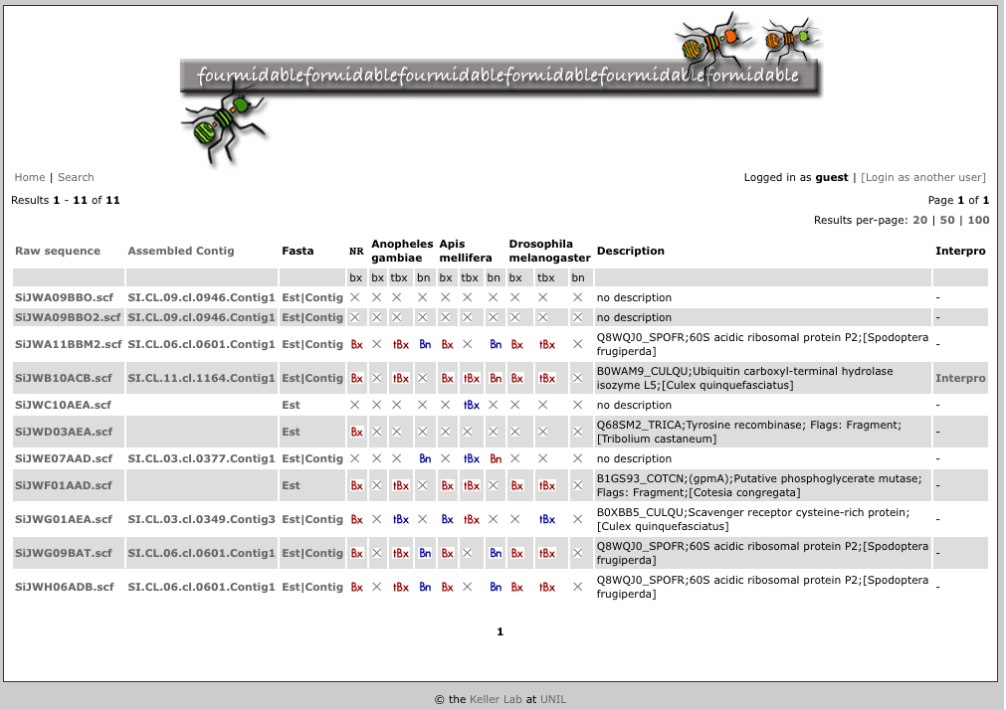
**Table of sequence search results**. For each result, the following are shown from left to right if applicable: sequence identifiers for raw and assembled sequence (these respectively link to the raw datafiles and assembly information); links to raw and assembled sequence in Fasta format; links to the results of BLAST against different databases (red buttons if E-value ≤ 10^-5^; blue buttons if 0.01 > E-value > 10^-5^; bx, tbx and bn respectively indicate BLASTX, TBLASTX and BLASTN algorithms); a description as inferred from BLASTX against the non-redundant protein database; and a link to Interproscan annotation.

Sequence searches result in tables with one line per sequence for easy access to sequence annotation (see Figure 1). In particular:

- Clicking on an identifier in the "Raw Sequence" column provides information on how that sequence was obtained and allows users to download the raw sequence. Tracefiles can be viewed with the Baylor College of Medicine Trace Viewer [29] or downloaded.

- Cleaned FASTA-format sequence can be downloaded for individual singlets and contigs.

- If the sequence is part of an assembled contig, the "Assembled Contig" identifier links to a display of the consensus sequence and the relevant input sequences as well as their quality scores. Additionally, a multiple sequence alignment highlights nucleotide polymorphisms within the consensus sequence.

- BLAST results between the sequence of interest and sequences from the non-redundant protein database and several insect nucleotide and protein databases are summarized by blue and red buttons, indicating weak (0.01 > E-value > 10^-5^) and stronger similarity (E-value ≤ 10^-5^) respectively. Clicking on a button displays the complete BLAST report.

- An additional link to Interproscan results and six-frame protein translations is displayed if Interproscan annotation is available.

### Additional Features

A convenient repository is available for ant molecular biology protocols (commonly in .doc or .pdf formats). New or revised protocols can be added via an upload form. Fourmidable also supports upload of result files from microarray gene expression experiments. The GEDAI platform allows straightforward sharing of microarray results and performing simple microarray analyses (including preprocessing, direct and indirect two-sample comparisons, 2 × 2 factorial and gene set enrichment analyses). GEDAI also provides summaries of the expression levels of specific microarray probe identifiers across multiple microarray experiments. Finally, Fourmidable provides download links to individual files containing all raw or assembled sequences, as well as sequence annotation in text format [see also Additional file 4].

### Past Applications

The sequence assembly and annotation information provided by Fourmidable has already proved useful in several published studies [14,30]. Most recently, Fourmidable's data helped J. Wang and colleagues to characterize genes that are differently expressed between workers from two alternative social forms of fire ants [6].

### Outlook

Fourmidable was initially developed as a private database in Lausanne. Recently it has been updated and made publicly accessible because of increasing interest in ant molecular research. To further develop Fourmidable, several primary investigators in the USA have submitted grant applications. This should lead to improved integration of gene expression data with sequence annotation, as well as support for genetic mapping and linkage data. When large amounts of genomic sequence become available for ants, the current approach for assembly and annotation may become computationally unrealistic. An alternative may be to adapt existing genome assembly, annotation and browsing tools.

## Conclusion

Fourmidable is a web-based database centralizing genomic resources for ants. As of October 2008, it contains raw sequence, assembled sequence, expression and annotation data for the fire ant *S. invicta *and the black garden ant *L. niger*, as well as ant-specific molecular biology protocols. Fourmidable will readily expand to accommodate additional data from these and additional species.

## Availability and requirements

Fourmidable is publicly available [27]. It has been tested with Firefox 2 and 3, Safari 3 and Internet Explorer 7. The web interface is valid HTML 4.01 Transitional and CSS 2.1.

## Authors' contributions

LF, PU, YW, FR, SJ and JW designed the database. YW, PU, FR and CI developed the database. LK supported the work. YW drafted the manuscript. LK, LF, JW and SJ revised the manuscript. All authors read and approved the final manuscript.

## Supplementary Material

Additional file 1 **Notes on implementation**. We provide several details about decisions made relative to the implementation of the database, and the assembly pipeline. Click here for file

Additional file 2 **List of tasks parallelized on the Swiss Institute of Bioinformatics Vital-IT computing cluster**. Some tasks were parallelized for increased execution speed. Click here for file

Additional file 3 **List of software parameters that differ from default**. For the assembly and annotation pipelines, default parameters were sometimes unsatisfactory. This table summarizes the parameters used when they differed from default. Click here for file

Additional file 4 **List of data available in text format**. Some of the data in Fourmidable can be downloaded in text format. Click here for file
