# Supplementary material for: Fourmidable: a database for ant genomics
Source: BMC Genomics. 2009 Jan 6;10:5. doi: 10.1186/1471-2164-10-5 (PMC2639375; doi:10.1186/1471-2164-10-5)
Supplement: Additional file 1 — Notes on implementation. We provide several details about decisions made relative to the implementation of the database, and the assembly pipeline. [file 1471-2164-10-5-S1.rtf]

Additional File 1: Notes on implementation.On assembly:In its current version, neither cluster nor contig identifiers generated during assembly are carried over from one assembly run to another. However, a tab-delimited text file is generated at the end of assembly run, showing correspondence between input sequences and contigs. Using this file, the history of which clones belong to which contig can be manually retraced. On database implementation:For each assembly “project” (meaning “species”), the MySQL database contains summary information referencing sequence, assembly, and annotations. Neither sequence nor annotation data are stored in the database, thus streamlining its size. Input data and cluster assembly information is stored in individual files; assembled sequence and blast results are respectively stored in indexed FASTA files and BLAST report files. All files are stored in project-specific directory hierarchies.Adding data for a new species is straightforward: a new species identifier is created in the database, and a new directory hierarchy is created on disk.
