# Supplementary material for: Fourmidable: a database for ant genomics
Source: BMC Genomics. 2009 Jan 6;10:5. doi: 10.1186/1471-2164-10-5 (PMC2639375; doi:10.1186/1471-2164-10-5)
Supplement: Additional file 2 — List of tasks parallelized on the Swiss Institute of Bioinformatics Vital-IT computing cluster. Some tasks were parallelized for increased execution speed. [file 1471-2164-10-5-S2.rtf]

Additional File 2: List of tasks parallelized on the Swiss Institute of Bioinformatics Vital-IT computing cluster.-	RepeatMasker-	CrossMatch-	Cap3-	Interproscan-	Blast searches	
