# Supplementary material for: Fourmidable: a database for ant genomics
Source: BMC Genomics. 2009 Jan 6;10:5. doi: 10.1186/1471-2164-10-5 (PMC2639375; doi:10.1186/1471-2164-10-5)
Supplement: Additional file 3 — List of software parameters that differ from default. For the assembly and annotation pipelines, default parameters were sometimes unsatisfactory. This table summarizes the parameters used when they differed from default. [file 1471-2164-10-5-S3.rtf]

Additional File 3: List of software parameters that differ from default.Program	Parameter	
SeqClean	-l 50	
RepeatMasker	-lib arthropodRepeats.fastathis “custom” repeats library is a download of all Arthropod and shared (ancestral) repeats from RepBase Update (23) in August 2008	
CrossMatch	-minscore 20-masklevel 100	
CAP3	-d 400-o 21-s 900	
BLAST (for clustering)	-p blastn -q -3 -G 2 -E 4 -K 0 -v 1 -b 1000 -e 1e-4	
BLAST (for annotation)	-v 200 -b 5 -e 0.01	
Interproscan	-trlen 30 -goterms -iprlookup -appl blastprodom fprintscan hmmpfam hmmsmart profilescan	
