# Supplementary material for: Fourmidable: a database for ant genomics
Source: BMC Genomics. 2009 Jan 6;10:5. doi: 10.1186/1471-2164-10-5 (PMC2639375; doi:10.1186/1471-2164-10-5)
Supplement: Additional file 4 — List of data available in text format. Some of the data in Fourmidable can be downloaded in text format. [file 1471-2164-10-5-S4.rtf]

Additional File 4: List of data available in text format. -	Raw, cleaned, and assembled sequences in FASTA format -	Assembly report-	BLAST results-	Gene Ontology annotation	
 
